# Creative Arts Interventions to Address Depression in Older Adults: A Systematic Review of Outcomes, Processes, and Mechanisms

**DOI:** 10.3389/fpsyg.2018.02655

**Published:** 2019-01-08

**Authors:** Kim Dunphy, Felicity A. Baker, Ella Dumaresq, Katrina Carroll-Haskins, Jasmin Eickholt, Maya Ercole, Girija Kaimal, Kirsten Meyer, Nisha Sajnani, Opher Y. Shamir, Thomas Wosch

**Affiliations:** ^1^Creative Arts and Music Therapy Research Unit, University of Melbourne, Melbourne, VIC, Australia; ^2^Health, Arts, Learning & Evaluation Lab, Department of Creative Arts Therapies, College of Nursing and Health Professions, Drexel University, Philadelphia, PA, United States; ^3^Music Therapy Lab, Faculty of Applied Social Sciences, University of Applied Sciences Würzburg-Schweinfurt, Würzburg, Germany; ^4^Theatre and Health Lab, Department of Music and Performing Arts Professions, Steinhardt School of Culture, Education, and Human Development, New York University, New York, NY, United States

**Keywords:** creative arts therapy, dance movement therapy, drama therapy, arts therapy, depression, older adults, processes, outcomes

## Abstract

Depression experienced by older adults is proving an increasing global health burden, with rates generally 7% and as high as 27% in the USA. This is likely to significantly increase in coming years as the number and proportion of older adults in the population rises all around the world. Therefore, it is imperative that the effectiveness of approaches to the prevention and treatment of depression are understood. Creative arts interventions, including art, dance movement, drama, and music modalities, are utilized internationally to target depression and depressive symptoms in older adults. This includes interventions led by trained arts therapists as well as other health and arts professionals. However, to date there has not been a systematic review that reports effects and examines the processes (why) and mechanisms (how) of creative arts interventions are used to address depression in this older age group. This systematic review of studies on creative arts interventions for older adults experiencing depression examined: outcomes of four creative arts modalities (art, dance movement, drama, and music); with particular attention paid to processes documented as contributing to change in each modality; and mechanisms considered to result from these processes. Our analysis of 75 articles (17 art, 13 dance, 4 drama, and 41 music) indicates mostly significant quantitative or positive qualitative findings, particularly for interventions led by creative arts therapists. Mechanisms of change gleaned from the studies that were common across modalities include physical (e.g., increased muscle strength; neurochemical effects, such as endorphin release), intra-personal (e.g., enhanced self-concept, strengthened agency and mastery; processing and communication of emotions), cultural (e.g., creative expression, aesthetic pleasure), cognitive (e.g., stimulation of memory), and social (e.g., increased social skills and connection), that were all considered to contribute to reduced depression and symptoms. Recommendations for future research includes stronger focus on testing of processes and mechanisms.

## Introduction

The number and proportion of older adults in the population has increased in virtually every country in the world over past decades, because of increased life expectancy and decreased fertility (United Nations Department of Economic Social Affairs Population Division, 2015). Current trends indicate an even greater global population of older people in coming years, with an estimated increase from about 12–22% (900 million to 2 billion) between 2015 and 2050 (Naghavi et al., 2015; World Health Organization, 2017b). Therefore, health issues impacting older adults are likely to make a significant contribution to the global health burden in coming decades (United Nations Department of Economic Social Affairs Population Division, 2017).

Depression affects approximately 7% of the world's older adults (World Health Organization, 2017a), with the highest rates in countries including Australia (10–15%) (National Ageing Research Institute, 2009) and USA (up to 27% with major depression and 31% with depressive symptoms) (Mental Health America, 2018). The most significant challenges are faced by older adults living in residential aged care, with rates as high as 35% (National Ageing Research Institute, 2009). Depression is three to four times more common in older people who have dementia (Bennett and Thomas, 2014). While prevalence is the same for both genders, functional disability causes by depression is greater for men (Forlani et al., 2014).

Depression is identified as the fourth leading cause of disability worldwide, and likely to be the second leading cause by 2020 (Murray and Lopez, 1996a,b). Depression leads to impaired functioning in daily life and can cause great suffering (Fiske et al., 2010). Depression also increases the perception of poor health, and the utilization of health care services and costs. Older adults with depressive symptoms have poorer functioning compared to those with other chronic medical conditions and higher rates of suicide. Mental health issues such as depression also impact physical health and vice versa (Bruce et al., 1994; Mental Health Foundation, 2018). Causes of depression are considered to include reduced involvement in daily life activities. This may be accompanied by self-critical thinking, which can exacerbate a depressed state. Protective factors relevant for depression in later life include age-related increases in psychological resilience, higher education and socio-economic status, engagement in valued activities, and religious or spiritual involvement (Fiske et al., 2010).

Documented depression treatments include pharmacological and non-pharmacological approaches. Pharmacological treatments indicate effectiveness in addressing symptoms of depression but are also associated with unwanted side effects (Beyond Blue, 2018; Department of Health, 2018). Psychology-informed approaches (behavioral therapy, cognitive behavioral therapy, cognitive bibliotherapy, problem-solving therapy, brief psychodynamic therapy and life review/reminiscence are indicated as effective (see for example, Thompson et al., 1987; Arean et al., 1993; Hsieh and Wang, 2003; Qualls and Knight, 2006). Preventive interventions including education, behavioral activation, cognitive restructuring, problem-solving skills training, group support, and life review have also received support (Fiske et al., 2010).

Creative arts (CA) modalities, including dance movement, drama, music, and visual arts, are also utilized internationally to target depression and associated symptoms. These include interventions by creative arts therapists (CAT) and by other therapists and health and arts professionals. While no systematic reviews have yet been published that examine research on depression and older adults across the CATs, previous reviews have been published for these modalities separately and for different age groups.

Two systematic reviews on art therapy (AT) and depression indicate that, for an all ages sample, art therapy has been utilized successfully (Blomdahl et al., 2013) and presents a cost-effective treatment model for mental health symptoms (Stevenson et al., 2015). In dance movement therapy (DMT), one systematic review of an all ages sample found that DMT may be beneficial for people experiencing depression, but without certainty because of small number of studies and low quality of evidence (Meekums et al., 2015). A meta-analysis of DMT studies including a sub-analysis for depression concluded that DMT may be effective in decreasing clinical symptoms (Koch et al., 2014). No systematic reviews on drama therapy (DT) for depression were found.

In music and music therapy (MT), a significant number of reviews indicate this modality's potential to support: reduced risk (Daykin et al., 2018); prevention (Sun et al., 2013); and decreased depression (Seinfeld et al., 2013; Chang et al., 2015; Innes et al., 2016; Travers et al., 2016; Zhao et al., 2016; Istvandity, 2017; Quach, 2017; van der Steen et al., 2017). Yet other music reviews reported no changes in depression (Ziv et al., 2008; Johnson et al., 2013; Vasionyte and Madison, 2013; Petrovsky et al., 2015; Xu et al., 2017).

A set of metaprocesses known as ‘common factors’ are understood to support growth and change through therapy (Ahessy, 2013). These common factors are largely informed by humanistic approaches to therapy which emphasize client-centered care (Rogers, 1951). They include therapeutic alliance, safety, empathy, inclusion, and unconditional positive regard (Wampold, 2001; Carr, 2008; Imel and Wampold, 2008).

While common factors are applicable to the creative arts therapies, there are additional processes employed in these approaches. Other therapies often target only cognitive processes, whereas the CATs seek to engage clients holistically across somatic, cognitive, emotional/intrapersonal, cultural (creative/aesthetic), and social/interpersonal aspects of the self. The integration of body and mind, or psyche, is a fundamental process of both DMT (Meekums, 2002; Sherwood, 2008) and AT, in which a “bodymind model” is proposed as a key contributor to change on a more meta level (Czamanski-Cohen and Weihs, 2016). In DT, core processes that reflect a holistic approach have been identified (Jones, 2007) along with metaprocesses (Cassidy et al., 2014) such as establishing safety, working in the here and now, being actively involved within or outside of the aesthetic frame, and working alongside the client while offering choice and control.

Adaptability is another technique employed in common by CA therapists, with methods, techniques, choices of medium and intervention styles adapted by therapists to best support the needs of each client or group, demonstrating responsivity to context and “attunement” (Kossak, 2009, 2015; Vermes, 2011; Holck and Geretsegger, 2016; Devereaux, 2017).

Finally, Koch (2017) has proposed key aesthetic processes that further distinguish the arts therapies from other approaches, such as intrinsic pleasure, authentic coherence, symbolism, transitional practices, and generativity. The application of processes that lead to and emerge from aesthetic expression are fundamental and most distinguishing of CAs from other types of therapy.

However, despite all this research, as yet there has not been a systematic review of the literature on the impact of CA interventions, both those that are led by CA therapists and other professionals, on depression and depressive symptoms in older adults and how these interventions are considered to work.

## Objectives and Research Question

This article offers a systematic literature review on the use of creative arts interventions to target depression and depressive symptoms in older adults. The article reports: outcomes of interventions across four creative arts modalities (arts, dance movement, drama, and music), for older adults experiencing depression or depressive symptoms; interventions implemented; and processes and mechanisms understood to contribute to therapeutic change.

## Research Question

Our overarching research questions are: What are the effects of creative arts interventions on depression or depressive symptoms in older adults? How are these interventions understood to work?

## Methods

### Study Design

#### Participants, Interventions, Comparators

This review examined studies about CA interventions, including CA therapy, intended to address depression or depressive symptoms, in older adults, across four modalities: art; dance; drama; and music; and combinations of these.

We examined studies utilizing all types of research methods and designs and did not specify particular comparators. We entered our review on the Prospero (International prospective register of systematic reviews) register (Centre for Reviews Dissemination, 2018) with registration number CRD42018091901.

### Systematic Review Protocol

#### Inclusion/Exclusion Criteria

This review included only articles that were:

- Published between 1.1.1997 and 1.2.18, in English language only;
- Of research, defined as investigation of a research question informed by quantitative or qualitative data, or both, including case studies and doctoral theses;
- Included only participants who were older adults (over 60 years, as per WHO definition) who had depression, as defined by DSM-V or ICD, and/or co-morbidity, or depressive symptoms with or without other disorders;
- Utilized a CA modality as an intervention to address such symptoms. This included CATs, which we define as interventions led by a CA therapist who were identified in the article as being trained or registered as a CAT in their specific modality, and other CA interventions led by other professionals;
- Locatable by the researchers.

### Search Strategy

We used the advanced search function on OVID, including databases: OVID Medliner (1946-present); CINAHL; EMBASE (Excerta Medica database); Medline; PsychINFO; Cochrane Central control trial register and Cochrane systematic reviews.

### Search Terms

The following search terms were used:

- “depression” or “depress^*^” or “MDD” (major depressive disorder); “LLD” (late life depression)
- AND “older adults” or “old^*^” or “gerontology” “geriatric^*^” or “late-life” or “aged care” or “aged” or “aging” or” “elderly”
- Art therapy: “art therap^*^” or “arts therap^*^” or “art psychotherap^**^” or “visual art therap^*^”
- Dance movement: “dance therap^*^” or “dance movement therap^*^” or “dance movement psychotherap^*^” or “dance/movement therap^*^” or “movement therap^*^” or “movement psychotherap^**^” or “dance” or “dance effectiveness” or “therapeutic movement”
- Drama: “drama^*^therap^*^” or “psychodrama” or “psychodramatic drama^*^therap^*^” or “process^*^ of change” or “applied drama” or “therapeutic theat^*^” or “improvi^*^ation” or “reminiscence theat^*^”
- Music: “song” or “guided imagery music” or improvi^*^ation” or song^*^writing” or “rap therap^*^” or “drum^*^” or “sing” or “choir” or “music” or “listen^*^”or “receptive music therap^*^.”

### Selection Process

The first search process, and round of decision-making concerning inclusion-exclusion was undertaken by a junior researcher with expertise in each field [authors KC-H (AT), ED (DMT), ME (DT), and JE (MT)]. The second process of assessment against the data extraction points listed below was undertaken by the same researcher, then cross-checked by a senior researcher from each field, authors: GK (AT); KD (DMT); NS (DT); KM (DT), TW (MT), and FB (MT). Where decisions between the two researchers were not concordant, discussion between them was undertaken to reach final agreement.

### Data Analysis and Quality Assessment

**Figure 1:**
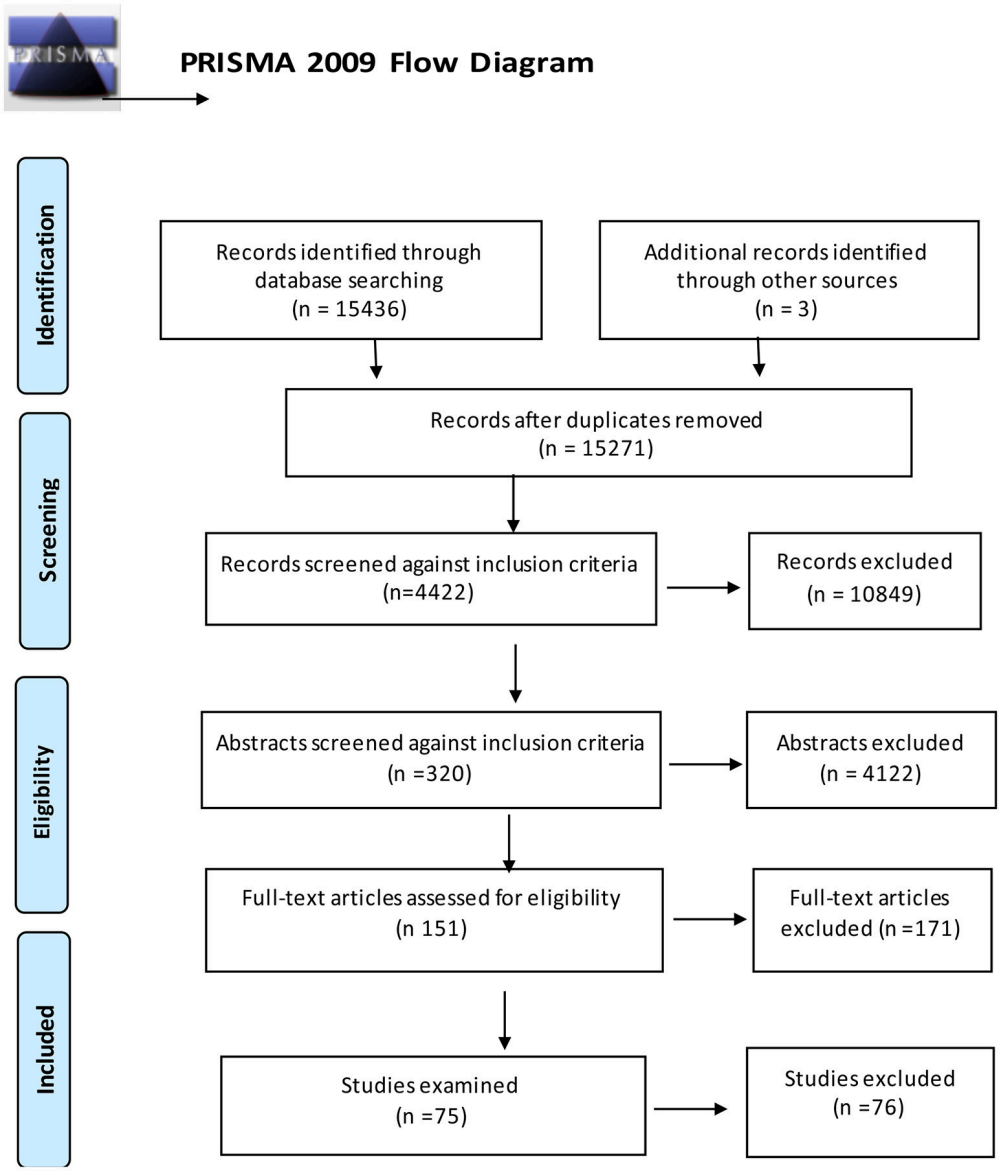
PRISMA flow diagram of studies retrieved.

Data analysis was undertaken in a staged process. First, all abstracts found through our search were considered against inclusion criteria listed above. This process is depicted in a PRISMA flow chart, provided in Figure 1.

Those fitting the criteria sufficiently were then analyzed and data extracted in the categories of: Participants—number and gender; Facilitator—training; Intervention—duration (number, length, and frequency of sessions), control/comparison, activities (what participants actually did); Outcomes—what and how assessed; Therapeutic techniques (defined as how the therapist operates in the intervention); Processes (defined as processes seen to elicit change in the client); and Mechanisms (what occurs within the client that results in change); and Study design. Articles were then assessed for quality using COREQ (qualitative studies) or PEDro tools (quantitative studies), or both tools (mixed-method studies). The COREQ is a formal checklist for evaluating the rigor and transparency of reporting in qualitative research, particularly for interviews and focus groups (Tong et al., 2007). The PEDro tool comprises elements agreed as suitable for quality assessment of RCT studies (Verhagen et al., 1998). One point was scored for each element of the study that met the criteria and these were tallied to arrive at a quality score for each study.

## Results

### Art Results

#### Summary of Studies and Quality Assessment

Seventeen art studies from an initial sample of 34 met all inclusion criteria as detailed in Table 1. Methodological approaches of these comprised: five qualitative studies, mostly individual case studies (de Guzman et al., 2011; Hoffmann, 2013) and interview methods; three mixed-methods; and nine quantitative studies, including comparisons of art therapy with other health interventions such as walking, music therapy, exercise, nature-based therapies, and community craft activities. Depression was addressed as a primary symptom (5/17); and as a co-morbid condition with Parkinson's disease (1/17), stroke (2/17), and dementia (2/17). Three studies focused on women because of the reported higher prevalence of depression with this population (3/17).

**Table 1:** Results of analysis of arts intervention studies.

**Studies**	**Interventions**	**Processes and mechanisms**	**Depression outcomes, measures and scales**	**Quality assess and design**
*Ali et al., 2014	“Group interactive art therapy” “drawing and painting using paper, pencils and crayons, making clay figures, drawing on an iPad, and taking photographs using a camera.” Group discussion about issues/emotional expression. *n* = 6 Session duration unknown; 2x week; 6 weeks	Non-directive approach with free use of art materials. Enjoyment of arts processes; experience of safe space where fears and concerns regarding experiences of illness could be expressed.	HADS Median score of 8 (borderline abnormal anxiety and depression) to median score of 6 (normal range) post-intervention.	COREQ 15/32 PEDro 3/11 MM, pilot study
+ Canuto et al., 2008	Group “encourages patients to express and understand emotions through artistic expression and creative processes.” Provides insight to emotions, thoughts, and feelings. *n* = 122 6 h; 2–3x week; duration unknown-ongoing during individual course of treatment.	Enhanced self-awareness and empowerment, self-esteem, reduced stress. Outcomes: improvement in mental quality of life, better adhesion to therapeutic community treatment and progress in patients' self-rating of group therapy	GDS (*p* < 0.001)	PEDro 7/11 LA
*Ceramidas, 2012	Group faith-based CBT intervention with art (abstract mixed-media paintings, clay to create animal forms/symbols of “life emerging,” individual collages depicting joys of life, religious symbols, and “free artwork,” found object collages created in pairs, and mask-making). “Encourage active, purposeful socialization and connectedness among residents.” *n* = 6 50 min; 1x week; 6 weeks	Observed outcomes: acceptance of the cognitive/physical limitations of others, seeking to understand others through requesting facilitator assistance, caring for others/including others during sessions, altruism, a sense of belonging, trust, humor, and spirituality.	GDS Not significant; Effect size not reported	PEDro4/11 PS, single group, pre and post-design
*Choi and Jeon, 2013	Group art therapy with collage medium and reminiscence therapy Facilitate recall of memories through engagement in collage making. Process to aid in facilitation of interpersonal engagement and memory recall to improve cognition. Facilitation of open dialogue among group participants. n = 66. 1 h; 2 x week; 5 weeks	Themes of support and empathy. Recall and sharing of memories- reflecting in group discussion on “the challenges posed by remembering,” “Internal integration” p. 329	GDS (*p* < 0.001)	PEDro7/11 QE
*Ciasca et al., 2018	Art therapy group with individual focus. Relaxation/guided imagery before art making. Verbal group processing post art making. Techniques involved themes leading to reflection on adaptation to difficult life circumstances, such as losses, death, finitude, resentment, solitude, and feelings of impotence. *n* = 56 90 min; 1x week; 20 weeks; n = 11	Reduction of anxiety, increased self-esteem. Shifting from passively waiting for guidance and assistance to increased independent engagement in art process. Relaxation, shifting from worried and “negative” thought patterns, feelings and emotions concretized through art.	GDS (*p* < 0.007) BDI (*p* < 0.025)	PEDro8/11 RCT
#de Guzman et al., 2011	Individual interviews and traditional Filipino art-making (TFA) “puni-making” to “overcome pangs of depression” through the “provision of figurative psychological crutches” to foster a positive view of life and self. *n* = 3 Length of intervention not specified.	Experience of support from researchers, feelings of accomplishment re ability to do something new and hopes to continue improving and learning new skills related to the art-making; engagement in new activities added to feelings of positive self-worth; engagement in traditional art-making offered opportunity to nurture self-esteem through exploring new skills and possibilities.	Emergent themes of depression and self-esteem: “Me, Myself, and Melancholy” and “Will Not Let My Worth Wither”	COREQ 13/ 32 QS phenomenological
*Doric-Henry, 1997	Individual pottery class/sessions based on Eastern Method throwing technique. Supporting and teaching participants ceramic process from start to finish, *n* = 40 (experimental group 20, control 20) 1 h; 1x week; 8 weeks	Increased self-esteem through mastery of materials.; shifting from passively waiting for guidance and assistance to increasingly independence in art process	BDI (*p* < 0.05)	COREQ 16/32 PEDro 5/11 QE
+ Drăghici, 2012	Group therapy; drawing tasks including draw a tree, house, silence, a rose bush, the ideal season, colors of life, draw feelings. Followed by verbal processing. Facilitating reminiscence, exploration of feelings, increasing self-insight/different aspects of self. Symbolization of strengths, weaknesses, and blockages. *n* = 30 (closed group 13, open group 17) unspecified session length; 8 sessions over one month.	Increased awareness of needs to resolve old conflicts, restoration of confidence, communication abilities, and feelings of belonging to a group	HDRS Effect size not reported	PEDro 3/11 PS, single group, pre and post- test design
+ Goldblatt et al., 2010	Group guided manipulation of modeling clay following protocol. No time limit for manipulating clay, but 12–40 min. followed by verbal processing. *n* = 22 One session (length unspecified)	Self-expression, autonomy, playfulness, and self-soothing. Reduction of ruminating and recurring thoughts” through guided clay manipulation and verbal processing.	BSI (*p* < 0.001)	PEDro 5/11 PS, single group, pre and post- test design
*Hoffmann, 2013	Individual intervention. Art therapy session directives: clock drawing test (CDT), Person Picking an Apple from a Tree (PPAT), a collage of likes and interests, non-directive modeling clay, family drawing, self-portrait, a wooden model airplane, and watercolor painting. *n* = 1 Session duration unknown; 1–2 x week; 6 weeks; 8 sessions total	Focus on creativity activity to switch focus from Parkinson's Disease (PD) to the task at hand, lessening stress levels/preoccupation with PD. Visual communication of thoughts/feelings	“Slight decrease in depressive symptoms” Parkinson's Disease Questionnaire (PDDQ-8) scores No effect size reported	PEDro1/11 SSR, single-subject research design with multiple baselines
#Hsu et al., 2017	Group drawing and painting tasks to *n* = 141 50 min; 1x week; 6 months	enable self-expression through different themes, Art therapy to reduce stress and incorporate fine motor skills/cognitive training	CSDD (*P* = 0.047)	PEDro5/11 RCS
*Im and Lee, 2014	Group session: mandala drawing, drawing taking turns, mud crafts, expression of body parts, collage, drawings of happy times in life. *n* = 94 (65 in art therapy, 29 in music therapy) 1 h; 1x week; 12 weeks	Promotion of autonomy and validation of experiences of disease/ depression/ negative experiences. “reduces the resistance of revealing and it also helps to the formulation of positive self-ego by respecting patients' imagination and unique personality.” Expressions of anger and hostility through visual arts, self-contemplation, expression of emotion in “socially acceptable ways,” use of imagination	KGDS (*p* = 0.000; *r* = 0.32)	PEDro5/11 PPT, one group
+Kang et al., 2010	Group session. Landscape composition technique (drawing a house, tree, and people), a squiggle drawing game, mixed-media collage (newspaper, cloth remnants, wallpaper, found items, etc.), mandala drawings, finger painting, molding clay, and drawings reflecting on themes of the past, present, and future. *n* = 38 (20 in experimental group, 18 in control) 3 h (30 min art therapy); 2x week; 9 weeks	Interventions developed to assess the mental state of the individuals who had difficulty expressing emotions, as a tool of expression, facilitated the individual's probing their own thoughts and feelings	Improved cognitive function, enhanced mental health, reduced depression KGDS (*p* < 0.001)	PEDro6/11 QE
*Kim H.-K. et al., 2016	Group and individual. 3 phases: traditional Korean art (1-10) Artmaking- structured art directives including pre-made clay figures that could be designed by participants, decorating bride and groom headpieces (11-25), creation of family photo frames, mandala drawings, and collaborative paintings (27-36) *n* = 28 (experimental group 14, control 14) 45 mins per session; frequency unknown; 36 sessions total	Development of rapport among group members, facilitate life review/reminiscence, self-integration, conflict resolution. Reduction in levels of depression and improved ability for self-expression	S-GDS (p = .036)	COREQ 4/32 PEDro 4/11 MM
*Kongkasuwan et al., 2016	Group session. 5 stages: meditation with music, warm-up activity, main activity (art-making) and group singing activity, ending with group-healing circle. *n* = 113 (59 in control, 54 in intervention) 1.5–2 h; 2x week; 4 weeks	“stimulate and benefit cognition, physical state, emotion, communication, social relations, and spiritual dimensions” improved concentration, emotion, self-confidence, and motivation;	HADS (*p* = 0.361)	PEDro8/11 RCT
*Lam, 2015	Group art therapy with movement, play, and music. Warm up prior to art making. Structured art directives followed by group processing . *n* = 11 2 h; 1x week; 12 weeks	Facilitation of meaningful group engagement, successful experiences in art processes, aiding in relaxation, increasing confidence and empowerment in ability to manage emotions. Increased socialization, increased aesthetic skills, increased-self-reflection, decreased depression, and anxiety, increased life satisfaction	GDS (*p* < 0.005)	COREQ 15/ 32 PEDro5/11 MM
*Rawtaer et al., 2015	Group session. Participants guided “through creative and narrative segments.” Specific interventions not stated. Multimodal approach of all included interventions emphasized. *n* = 101 30 min; 1x week; 10 weeks 30 min; biweekly; 42 weeks; 1-year total	Mental stimulation and social/group engagement.	Reduce negative emotions and anxiety and improve self-esteem. SDS (*p* < 0.05)	PEDro6/11 OS

***Key:** Depression outcomes: We use the term ‘outcomes’ here for all studies, even though only some studies offer calculations of effects that adjust for outcomes compared to control group*.

*BDI, Beck Depression Inventory; BSI, Brief Symptom Inventory; CSDD, Cornell Scale for Depression in Dementia; GADI, Goldberg Anxiety and Depression Inventory; GDS, Geriatric Depression Scale; HADS, Hospital Anxiety and Depression Scale; HDRS, Hamilton Depression Rating Scale; KGDS, Short Form of the Korean Geriatric Depression Scale; S-GDS, Short Geriatric Depression Scale; SDS, Zung Self-Rating Depression Scale*.

**Intervention coding**: * intervention delivered by trained or registered art therapist; # intervention delivered by other professional; and + unclear.

***Design codes**: COD, crossover design; CS, case study; CDA, collaborative discourse analysis; LA, longitudinal study; MM, mixed methods; OS, observational study; PE, pre-experimental (i.e. a single group studied without comparison to control group); PPGD, prospective, parallel-group design; PPT, pre and post-test; PS, pilot study; PT, pragmatic trial; QE, quasi experimental; QS, qualitative study, RCT, randomized control trial; RCS, retrospective cohort study; SSR, single subject research*.

Qualitative studies scored between 1 (2/5) and 8 (1/5) out of 11 on PEDro. The limitations of these studies included inadequate reporting details in data collection and lack of rigor in measures to ensure credibility in data analysis. Quantitative and mixed method studies scored between 4 (1/12) and 16 (2/12) out of 32 on COREQ. The main shortcomings of these studies were that samples were not randomized, blinded, or adequately powered. They tended to be small in scope and lacking rigorous efforts to ensure validity in the findings. The majority did not include adequate details on method to enable replication. No art studies reported any results from follow up.

Eleven/Seventeen studies were led by an art therapist, 2/17 by another professional; and 4/17 had a leader of unknown training. Twelve studies had significant findings: 8/12 led by an art therapist, 1/12 by another professional and 3/12 by a leader of unknown training.

### Interventions

Art programs were typically sessions of an hour, held once or twice a week over periods from 12 to 52 weeks. Interventions were typically offered as group format, with specific attention provided by therapists to individual group members. Two interventions were one on one programs (de Guzman et al., 2011; Hoffmann, 2013).

Art media options and activities included traditional crafts and arts (de Guzman et al., 2011; Ciasca et al., 2018) and clay and painting. For example, in one task, participants manipulated clay into a ball, divided it into parts and re-assembled it into another object, then shared their experiences in verbal discussion (Goldblatt et al., 2010). Choices of media were frequently discussed as important, particularly because of challenges identified with patients' physical mobility and fine motor control.

### Therapeutic Techniques

The therapeutic techniques reported in the art studies included the therapist being encouraging of participants' expression and learning through art making, sensitive to individual needs, encouraging of interaction and pro-social experiences between group members.

### Proposed Mechanisms of Change

Based on the processes identified in the literature, we propose that the mechanisms of a change for depressive symptoms through art interventions are:

- Physical: engagement in a creative activity that had physical aspects was seen to catalyze relaxation and reduction of stress (Canuto et al., 2008; Goldblatt et al., 2010; Lam, 2015; Hsu et al., 2017)
- Cultural: the making of art was seen to facilitate creative expression and play (de Guzman et al., 2011; Kim H.-K. et al., 2016); the use of context-responsive creative expression was seen as significant; creative expression was enabled by use of accessible media of clay and painting (Goldblatt et al., 2010); evocation of familiarity and positive memories was catalyzed by use of culturally appropriate traditional crafts and arts (de Guzman et al., 2011; Ciasca et al., 2018)
- Emotional/intrapersonal: creation of art products was seen to provide valuable distance, and enable externalization and visual communication of inner subjective experiences (Goldblatt et al., 2010; Kang et al., 2010; Drăghici, 2012; Choi and Jeon, 2013; Ali et al., 2014; Im and Lee, 2014; Hsu et al., 2017; Ciasca et al., 2018); expression of positive and negative emotions (Kang et al., 2010; Ali et al., 2014; Im and Lee, 2014; Kim H.-K. et al., 2016; Kongkasuwan et al., 2016); promotion of autonomy (Doric-Henry, 1997; Canuto et al., 2008; Goldblatt et al., 2010; Ceramidas, 2012; Kongkasuwan et al., 2016); and positive views of self (Canuto et al., 2008; de Guzman et al., 2011; Drăghici, 2012; Rawtaer et al., 2015; Kongkasuwan et al., 2016; Ciasca et al., 2018); agency and mastery was seen to be strengthened by the act of completing an art piece (Doric-Henry, 1997; de Guzman et al., 2011; Hoffmann, 2013; Lam, 2015; Ciasca et al., 2018)
- Cognitive: art-making was seen to enable: reinforcement and recall of positive memories (Canuto et al., 2008; Choi and Jeon, 2013; Hoffmann, 2013; Kim H.-K. et al., 2016); addressing of concerns around death, loss and end of life (de Guzman et al., 2011; Ali et al., 2014); and distraction from ruminative thoughts (Goldblatt et al., 2010; Ciasca et al., 2018)
- Interpersonal: group work (with or without therapist's involvement) was seen to encourage socialization and sharing (Canuto et al., 2008; de Guzman et al., 2011; Ceramidas, 2012; Drăghici, 2012; Choi and Jeon, 2013; Ali et al., 2014; Im and Lee, 2014; Lam, 2015; Rawtaer et al., 2015; Kim H.-K. et al., 2016; Kongkasuwan et al., 2016).

### Dance Movement Results

#### Summary of Studies and Quality Assessment

Thirteen studies met all inclusion criteria from an initial sample of 29 as detailed in Table 2. Studies were predominantly RCTs (6/13); with quasi-experimental (3/13); and single condition over two or three time periods (4/13). Only one of the 13 studies was focused on depression as a primary diagnosis, with the other 12 addressing depressive symptoms.

**Table 2:** Results of analysis of dance movement intervention studies.

**Studies**	**Interventions**	**Processes and mechanisms**	**Depression outcomes, measures and scales**	**Quality assess and design**
# Adam et al., 2016b Status	Dance and relaxation: warm up; poco-poco dance, relaxation incorporating progressive muscle relaxation; group sessions. *n* = 44 60 min; 2 x week; 6 weeks	No mechanism discussed specifically, but implied relationships between physical and cognitive improvement through dance leading to reduced depression. Suggested: Better scores for women perhaps because dance-like activities more attractive to females	HADS (*p* < 0.000)	PEDro 2/11 QE
# Adam et al., 2016a Effectiveness	Dance and relaxation exercises: warm-up and stretching activities followed by poco- poco dance session in group session; *n* = 84 (44 intervention, 40 control). 60 min; 2x week; 6 weeks	No mechanism discussed specifically, but relationships between other outcomes noted including: improved QOL, increased cognitive and physical function and enhanced wellbeing; enhanced coping and increased sense of independence. Stimulation of the parietal lobe through dance provides somatosensory input that may increase the neurotrophic factor that improves cognitive and visuospatial function. Non-competitive type of dance may make it more favored by participants. Therapeutic benefits are motivating for adherence.	HADS (*P* < 0.001)	PEDro 6/11 QE
+ Alpert et al., 2009	Modified age appropriate jazz dance class; in group sessions. *n* = 13 Session duration unknown; 1x week; 15 weeks	Relationship between physical activity improving balance and other physical skills in social setting and mood postulated. Its inherent “fun” factor may contribute to adherence and success.	GDS, not significant	PEDro 4/11 PS single condition, three time periods
# Britten et al., 2017	Modified contemporary dance program: warm up; basic low impact aerobic movements; series of moves; improvisation; cool down (breathing and stretching exercises); group session. n = 22 90 mins; 2 x week; 8 weeks	Participants perception of benefit of physical activity, motivation provided by the group context; psychological benefits such as use of brain, improved mental health. Cognitive health stimulation posited as important; creative and didactic elements Valued; seen as suitable mostly for women	GDS (*p* < 0.05)	PEDro 4/11 PPT uncontrolled ‘pre-post’ intervention; focus groups
# Cross et al., 2012	live dance performance and receptive music listening; n = 100 (50 in dance intervention); group intervention (participants- audience members) 30 min x 1	Possible factors but no hard evidence of viewing dance eliciting positive memories, enjoyable aesthetic experience, communicating something of meaning	BDI (t49 = 11.95, *p* < .001, *d* = 0.88).	PEDro 7/11 RCT
# Eyigor et al., 2009.	Dance-based exercise program: a warm-up, folklore dance stepping, stretching, cool-down. participants in circle; group sessions; n = 37 (dance intervention 19 females). 1 hour; 3 x week; 8-weeks	Age appropriate dance moves; strong focus on following choreography and improving physical capability and functional mobility; dance is pleasurable and may motivate other activity; group increased motivation; folkloric dance rhythms appropriate for age group and cultural background	GDS, no significant improvement, but verbal expression that they felt happier after the dancing exercise	PEDro 7/11 RCT; interviews
+ Garcia Gouvêa et al., 2017	Dance classes: low impact choreography; warm ups; sitting waltz/standing waltz, gentle movement, stretching; group sessions; n = 20. 45 min; 3x week; 3 months (total 40 classes)	Rhythmic moves to improve functional, emotional and behavioral skills; integration of physiological, psychological, sociological aspects of wellbeing; facilitate self-expression and communication; reduced fear and isolation, and better self- esteem; memorization of movement sequences and attention supported by intentional changes of movements make high cognitive demand that can help reduce depression	BDI, no significant improvement	PEDro 3/11 PE pre-experimental, pre and post-test, convenience sample
# Haboush et al., 2006.	Ballroom dance lesson weekly: foxtrot, waltz, rumba, swing, cha-cha, and tango; one on one session; n = 20. concern, empathy 45-min; 1x week; 8-weeks	not psychotherapy, but some common factors of therapy present including concern, empathy, a treatment setting, a therapeutic procedure; pleasure in learning; exercise and enjoyment of music.	HDRS (d = .51) and GDS (d = .40) medium range; interview	PEDro 8/11 RCT
# Jun et al., 2013	Music-movement therapy (MMT): preparatory activities (movement exercises with quiet meditational music): main activities, finishing activities; group sessions; n = 40 (dance intervention 20). 60 min; 3x week; 8 weeks	No explanation.	CES-D, no significant difference	PEDro 6/11 RCT, QE, with pre- and post-tests
# Matto et al., 2015.	Listening to music; imagery activation; body movement; sharing experience with others; group theme; group sessions; *n* = 20 (92% female) 50–60 min, each group; 10 weeks; unclear number of times per week.	Hypothesized effect: creative arts participation enhances positive social engagement, which enhances mood; enjoyment, opportunities for behaviors such as remembering, recognizing, and expressing what they were feeling, and understanding, appreciating, and being sensitive to others	GDS-SF, not significant	PEDro 8/11 RCT, interviews
# Murrock and Graor, 2014.	Dance class; 5-min warm-up, 30 min of simple low intensity dance steps, 10-min cooldown; group session; *n* = 20 Dance steps adjusted to cater for physical capacity; onsite intervention; non-judgemental attitude; provision of chair 45 min; 2x week; 12 weeks	increased physical function; social interaction; altered mood, increased learning	CES-D, *t* = 6.11, *p* < 0.001, r|2 = 0.65,	PEDro 5/11 PPT One-group, pretest and posttest
# Sun et al., 2013	Multi-modal approach “mind–body meditative approach” (MBMA) Tai Chi exercise, dancing, (Chinese cultural and Latin dancing), playing musical instruments, choral singing, and operas, formal meditative practice. (awareness of breathing, awareness of emotions, control of emotions and concentration); group sessions; *n* = 750 2.5 h, weekly, 15 months.	Tai chi appealing to older people. Dancing, in combination with Tai chi and singing, may protect from metabolic syndrome and brain function decline, and promote a positive QoL including psychological health; social engagement and ability to cope well with depression; enjoyment; dance seen as attractive to women. Based in community and natural environment, did not focus on the disease and was friendly and socially comfortable, thus enjoyable and sustainable.	GHQ30 significant, dancing groups had the lowest depression rate among the four interventions	PEDro 6/11 RCT, case controlled design
# Vankova et al., 2014	Exercise dance: warm-up: slow-paced leg and arm movements seated; main period of ballroom dance, including foxtrot, waltz, cha-cha, cancan; cooldown used relaxation techniques, deep breathing, stretching; group sessions; *n* = 162, mostly women. Adaptation of exercises; goal to make class enjoyable 60 min; 1x week; 3 months.	The characteristics of the traditional dance and reminiscence; of the proven relationship between depressive symptoms and functional movement status; group intervention provides participants with the opportunity to do something together and share the experience; interaction withpeers could lead to increased self-confidence and feeling of competency	GDS, *P* 0.005	PEDro 9/11 RCT

***Key:** BDI, Beck Depression Inventory; CES-D, The Center for Epidemiologic; Studies Depression Scale; CSDD, Cornell Scale for Depression in Dementia; GADI, Goldberg Anxiety and Depression Inventory; GDS, Geriatric Depression Scale; GDS-SF, Geriatric Depression Scale short form; GHQ30, General Health Questionnaire; HADS, Hospital Anxiety and Depression Scale; HDRS, Hamilton Depression Rating Scale*.

**Intervention coding:** * intervention delivered by trained or certified dance movement therapist; # intervention delivered by other professional; and + unclear.

***Design codes:** COD, crossover design; CS, case study; CDA, collaborative discourse analysis; LA, longitudinal study; OS, observational study; PE, pre-experimental; PPGD, prospective, parallel-group design; PPT, pre and post-test; PS, pilot study; PT, pragmatic trial; QE, quasi experimental; RCT, randomized control trial; RCS, retrospective cohort study; SSR, single subject research*.

Quality scores using PEDRO were evenly spread between 2 and 9 out of 11. Eleven of these studies were led by another professional; two by a leader of unknown training; and none by a dance movement therapist. This was despite several studies stating that the intervention was dance movement therapy, while providing no indication that a certified DM therapist was involved. One study (Cross et al., 2012) claimed to be DMT and included information about DMT in the literature review, but then described an intervention of clients watching professional dancers that seemed to have no relationship at all with DMT principles. In fact, no studies involving dance movement therapy were included in our final sample. Ten studies had significant findings: nine led by professional who was not a dance movement therapist, and one by a leader of unknown training.

Limitations of the dance studies included small sample sizes, with almost half having an n of 20 or less. Low PEDro scores was mostly caused by lack of concealment of allocation, lack of blinding of participants, therapists and assessors, and lack of random allocation. Another limitation was that few studies (4/13) included a mention of follow-up component. One study (Vankova et al., 2014) mentioned an improvement in GDS scores in one follow up (5.0–4.5), however, this data was not included in the results. Another study (Matto et al., 2015) reported follow up results indicating a decrease in GDS scores (2.44–2.19), supported by interviews with participants about their enjoyment in being part of the group where self-expression and understanding, appreciating and being sensitive to others was a commonly valued experience (p. 280). Two other studies mention a follow up but do not report results (Haboush et al., 2006; Jun et al., 2013).

### Interventions

Programs offered were typically sessions of between 30 and 90 min, with 60 min being most common; held between one and three times weekly, most often twice; for periods from one week to 15 months, most often 8 weeks. Interventions were typically offered as group sessions, with specific attention provided by trained therapists to individual group members. One study described a one on one program. Interventions were primarily focused on the acquisition and repetition of dance steps, including western dance styles such as jazz, contemporary and ballroom, as well as traditional and folk dances. Session structure most frequently was a brief warm up, then teaching of dance steps, followed by a final cool down which sometimes included relaxation.

### Therapeutic Processes

The dance studies did not include much discussion about therapeutic processes, perhaps because none of the studies examined was led by a dance movement therapist. This was in contrast to other modalities where the majority of studies involved CA therapists. There was a strong emphasis on physical outcomes, particularly in the area of functional mobility. This was presumably because the studies were mostly facilitated by dance instructors and physical therapists who emphasized functional performance as a primary concern, which they addressed through programs that involved the acquisition of dance skills. In several studies, changes in clients' physical state through dance, such as improved balance and strengthened muscles, was seen to be related to reduced symptoms of depression, even though this had not been the central focus of the intervention. For example, Britten et al. (2017) reported reduced falls-risk as a result of dance participation, which was seen in turn to have positive impact on mood states such as depression. However, causal pathways were generally not articulated clearly or trialed in these studies.

Several studies included comment on the greater acceptance of dance-based interventions by women than men, thus explaining more successful engagement, stronger adherence, and better outcomes of female participants in many interventions.

### Proposed Mechanisms of Change

Based on the processes identified in the literature, we propose that the mechanisms of a change for depressive symptoms through dance interventions are:

- Physical: improved physical performance and function including balance, muscle strength, joint sense and proprioception;
- Cultural: enjoyable aesthetic experiences;
- Cognitive: cognitive decline slowed through exercise and stimulation of brain circuits used to learn dance steps; activation of motor neurological brain regions through improvised or expressive movement that may contribute to changes in brain structure;
- Social: positive social engagement, stimulation and enhancement of communicative and relational capacities through shared experiences in dance.

### Drama Results

#### Summary of Studies and Quality Assessment

Four studies met all inclusion criteria from an initial sample of 25 as detailed in Table 3. Methodological approaches included one quasi-experimental study (Keisari and Palgi, 2017), one mixed method (Wilkinson et al., 1998), two qualitative studies employing a combination of ethnographic observation, qualitative interviews, and practice reflections (Kontos et al., 2017) and one collaborative discourse analysis (Sajnani et al., 2018). One study addressed depression as the primary diagnosis (Keisari and Palgi, 2017), while the other three addressed depressive symptoms.

**Table 3:** Results of analysis of drama intervention studies.

**Studies**	**Interventions**	**Processes and mechanisms**	**Depression outcomes, measures, and scale**	**Quality assess and design**
Keisari and Palgi, 2017	Life review of major life crossroads integrated with drama therapy techniques led by drama therapist and social worker used dramatic roles, embodiment, enactment, witnessing; *n* = 55 (27 intervention, 28 control). Group intervention 90 min; 1x week; 12 weeks	Increased self-acceptance, meaning making, relationships with group members, capacity for self-reflection and the integrity and coherence of one's life story. Social processes such as social recognition, learning from others, and being able to help others that are central to life review were amplified through use of processes of dramatic projection, embodiment, enactment, witnessing, and the life-drama connection.	GDS: [*F*_(1, 53)_ D 12.9, *p* < 0.001], Significant improvement reported in depressive symptoms	PEDro 9/11 QE
Kontos et al., 2017	Improvisational play, humor, empathy, song, dance, and music facilitated by trained elder clowns; *n* = 23. Individual intervention 10 min; 2x week; 12 weeks	Reciprocal play, affective relationality, joy, con-constructed imagination, and acceptance of sadness as non-pathological all regarded as contributing to positive affect in participants.	Qual interviews, ethnographic observations of video recorded clown- resident interactions, and practice reflections	COREQ 21/32 QS
Sajnani et al., 2018	Use of projective devices, psycho-dramatic, techniques drawn from Therapeutic Spiral Method (TSM), and sociodrama. Group and individual intervention *n* = 4 Average 45 min; 1 X week	Reinforcing participants' strengths, dramatic projection prompts social interaction and facilitates perspective through externalization of inner conflicts.	Collaborative discourse analysis	COREQ 19/32 CDA
Wilkinson et al., 1998	Sesame method of DT led by drama therapist included use of metaphor, movement, enactment. *n* = 16 (9 intervention, 7 control). Group intervention 105 min; 1x week; 12 weeks	Stimulation of memory, encourages role flexibility, and reinforces past coping strategies. Metaphor encourages organization of self-expression. Enactment of scenes increases orientation to past and present, self-understanding, and acceptance, meaningful personal relationships.	CSDD Qualitative data involving observation and informal interviews. No significant differences reported between intervention and control group with exception of indication of deterioration in area of depression due to rise of depressive symptom in one participant	PEDro 8/11 QE/ QS

***Key:** CSDD, Cornell Scale for Depression in Dementia; GDS, Geriatric Depression Scale*.

**Intervention coding:** * intervention delivered by a trained, registered and/ or certified drama therapist; # intervention delivered by other professional; and + unclear.

***Design codes**: CDA, collaborative discourse analysis; COD, crossover design; CS, case study; LA, longitudinal study; OS, observational study; PE, pre-experimental; PPGD, prospective, parallel-group design; PPT, pre and post-test; PS, pilot study; PT, pragmatic trial; QE, quasi experimental; QS, qualitative study; RCT, randomized control trial; RCS, retrospective cohort study; SSR, single subject research*.

The two studies employing quantitative methods scored between 8 and 9 out of 11 on PEDro. The limitations of these studies were that samples were small and not randomized or blinded. The two qualitative studies met between 19 and 21 elements of 32 in COREQ. The limitations of these studies included inadequate reporting of details in data collection, and a lack of reported efforts to increase the trustworthiness of findings. The main shortcoming overall was the lack of detail on the method and activities used that would enable replication.

Two interventions were led by a drama therapist, one by a drama therapist with another professional, and one by other professionals (elder clowns). Of the studies involving drama therapists, one reported significant quantitative findings and two reported no statistically significant findings but positive qualitative outcomes. The study led by elder-clowns reported positive qualitative outcomes. No follow up data was provided for any of these studies.

### Interventions

Programs offered by drama therapists were typically group sessions of between 45 and 105 min for an average of 80 min, held once weekly, for 12 weeks. One study facilitated by elder clowns involved two clowns per individual resident for 10 min twice weekly. Interventions involved therapists' technique of empathic attunement, and activities involving mirroring, doubling, and role-reversal, dramatic embodiment of inner conflicts, dramatic projective in the form of roleplay and enactment. Session structure in group interventions typically involved a warm up, a main action and a closure phase. One on one sessions conducted by elder clowns involved techniques of affective attunement and humor, and activities involving reciprocal play.

### Therapeutic Processes

Each study that included drama therapy involved therapeutic techniques derived from meta-processes (Cassidy et al., 2014) including the therapist being involved while working in the here and now alongside clients, the establishment of safety through the choice of techniques and clients being offered control and choice to enable them to exercise initiative and creativity. Other core processes, as identified by Jones (2007), included play, dramatic embodiment, dramatic projection, personification, and impersonation (role-play), empathy and distancing, life-drama connection, witnessing, and transformation. These were used to facilitate clients' short and long term goals. For example, in the study involving drama therapy and life review, stories of group members were embodied by other group members who took on the roles identified in the single group member's story and created dramatic images. This was intended to enable the story owner to “gain new perspectives about their life decisions and deepen their understanding of the associations between these decisions, in a way that gives rise to a more positive identity” (Keisari and Palgi, 2017, p.1080).

The core processes used to activate internal resources and externalize internal conflicts were discussed by Sajnani et al. (2018). Wilkinson et al. (1998), reported activities being chosen to stimulate reminiscence, socialization, and “provide opportunities for more organized self-expression through the use of metaphor” (p. 195). In the study in which elder clowns interacted with older adults, the primary processes described were (a) affective relationality; (b) reciprocal playfulness; and (c) co-constructed imagination.

### Proposed Mechanisms of Change

The studies reviewed suggest that shifts in depressive symptoms that result from drama interventions are catalyzed by mechanisms of change such as:

- Physical: engagement in playful, embodied activity contributing to sense of vitality and regulated, relaxed breathing;
- Cognitive: orientation to past and present, reinforcement of positive coping strategies, coherent organization of self-expression, increased memory recall, facilitation of meaning making;
- Emotional/intrapersonal: use of metaphors, roles, and playful, embodied enactments providing a suitable distance to activate internal resources and externalize and communicate inner conflicts and strengths, and facilitate emotional regulation;
- Social: individual and group activities prompting increased positive social interaction.

### Music Results

#### Summary of Studies and Quality Assessment

Forty-one studies met all inclusion criteria from an initial sample of 91 as detailed in Table 4. Depression was addressed as a primary diagnosis in 21 studies and as secondary diagnosis and depressive symptoms in 20 studies.

**Table 4:** Results of analysis of music intervention studies.

**Studies**	**Interventions**	**Processes and mechanisms**	**Depression outcomes, measures and scales**	**Quality assess and design**
* Ashida, 2000	Familiar songs in small groups, *n* = 40 38–45 min, 5x/week, 3 weeks	Improvement of mood and interaction skills	CSDD (*p* < 0.05)	PEDro 6/11 COD
# Berger et al., 2004	Active and receptive music therapy (singing, listening preferred music, playing, reminiscence, movement to music), *n* = 72 60 min, 1x/week, 24 months		no significant difference in depression (BDI *p* 0.27-0.73, GDS *p* = 0.31–1.00), trend higher depression in control group month 12, and higher depression in month 24	PEDro 11/11 RCT/PT, two-arm
# Brandes et al., 2010	Online individually calibrated music listening: MT1-program (composed polyphonic modern music), MT2-program (classical music individually calibrated), *n* = 204 30 min, daily, 4*5weeks	Neurophysiologic and neurochemical effects of receptive music therapy	MT1-program HAM-D (*p* 0.013), BDI (*p* 0.361), HADS-D (*p* 0.014), COMP (*p* 0.030) MT2-program HAM-D (*p* 0.031), BDI (*p* 0.030), HADS-D (*p* 0.024), COMP (*p* 0.059)	PEDro 11/11 RCT/PT, two-arm
* Castelino et al., 2013	Group music therapy, improvisation, pre-recorded music for coping anxiety and depression, *n* = 40 60 min, 1x/week, 10 weeks		HDRS (*p* 0.02)	PEDro 5/11 COD
# Chan et al., 2009	Instructed four types of music-listening, each day home-training in evening of participant him-/herself, *n* = 58 30 min, 1x/week, 4 weeks	processing music stimuli in rhythm and pitch and limbic system (neuropsychological effects)	GDS (*p* < 0.001)	PEDro 10/11 RCT/PT, two-arm
# Cheung et al., 2018	MM (welcome + closing song + MM: batting balloons, waving ribbons, foot tapping, playing musical instruments, mimicking movements demonstrated by interventionist.) vs. music listening vs. social activity, *n* = 165 30 min; 2x week; 6 weeks	Expressive and relational abilities that promote new learning strategies and improve well-being	GDS (*p* 0.02), reduced depressive symptoms [*F*_(4, 324)_ D 2.51, *p* D.042, partial ?2 D 0.03)	PEDro 10/11 RCT, multi-center
+ Chiung-Yu et al., 2016	Stimulative + sedative music videos, *n* = 30 30 min, 1x/week, 2 weeks		GDS (*p* 0.001–0.03)	PEDro 10/11 COD / CS
* Chu et al., 2014	Gross/fine motor movements in music, rhythm playing, listening to popular music, rhythm playing with instrumental accompaniment, singing with instrumental accompaniment; music-prompted reminiscence, *n* = 104 30 min, 2x/week, 6 weeks		CSDD (*p* = 0.001)	PEDro 11/11 ṟeak PPGD, permuted-block randomization
# Coulton et al., 2015	Singing group guided by “Sing for your Life”-trained facilitators, *n* = 258 90 min, 1x/week, 14 weeks	Enjoyment of the experience (self-report)	HADS (*p* < 0.01)	PEDro 10/11 RCT/PT, two-arm
# Cross et al., 2012	Listening to pre-recorded music, *n* = 100 30 min, one time	Connection with music and movement improves decrease of depression	BDI: 3 days: *t*_(49)_ = 6.34, *p* < 0.001, d = 0.61, 10 days: t_(49)_ = 4.60, *p* ≤ 0.001, d = 0.44	PEDro 8/11 PPT, two-arm
* de la Rubia Ort et al., 2018	Welcome + theme songs, n = 25 60 min, one time	Welcome song: activate cognitive area, improving recent memory, remembering the names; + theme song: related to flowers, attention focusing on musical task, lyrics, visual agnosia: recognition of faces / band members + of the day of the week.	HADS (*p* 0.001)	PEDro 5/11 QE, analytical, prospective study
*Elefant et al., 2012	Group music therapy with breathing, vocal and singing exercises, *n* = 10 60 min, 1x/week, 20 weeks	Singing may facilitate a relaxation response which directly increases vocal fold flexibility, enabling the speaker to express more emotional dynamics in his voice	MADRS no significant change	PEDro 11/11 PT, two-arm
# Fancourt et al., 2016	Drumming sessions, *n* = 45 90 min, 1x/week, 10 weeks	Social element of group drumming, activating	HADS-D (*p* < 0.001)	PEDro 11/11 PT, two-arm
# Giaquinto et al., 2006	Group singing accompanied by guitar (of music teacher and nurse), *n* = 12 45 min, 6x/week, 2 weeks	Military marches, 4/4 time	HADS (*p* 0.014)	PEDro 8/11 PS, cross over design
* Giovagnoli et al., 2017	Active music therapy, *n* = 33 45 min, 1x/week, 12 weeks	Triggered emotion and interpersonal relationships	BDI (*p* = 0.017)	PEDro10/11 RCT, single-blind
* Gök Ugur et al., 2017	Listening to folk/instrumental songs, picked before session, *n* = 64 40 min, 3x/week, 8 weeks	Music increases the independence feeling, self-confidence; leads to cope with feelings, such as helplessness and depression; induce alpha waves; trigger the endorphin release	GDS (*p* 0.006)	PEDro10/11 RCT, single-blind trial
+ Gopi and Preetha, 2016	Music listening, *n* = 30 30 min, daily, 15 days	Music affects mood, feelings, physiological functions; accesses deep emotions	GDS (*p* 0.01)	PEDro 5/11 QE
# Guétin et al., 2009	Special individual receptive music therapy (questionnaire individual preferences and experience, computer modifying program of music for special 20 min dynamic), *n* = 38 20 mins, 1x/week, 16 weeks	Receptive stimuli of this method stimulates cognitive functioning “to recall autobiographical memory and images”	GDS (*p* < 0.05 w4, *p* < 0.01 w8-16, *p* < 0.05 w24; persistence depression *p* < 0.003)	PEDro 10/11 RCT/PT, two-arm
# Han et al., 2011	Music therapy and activities program, also physical exercise and cognitive stimulation, *n* = 45 6 h, 1x/week, 8 weeks	Cognitive and physical activities, social participation, capabilities	Revised Memory and Behavioral Problems Checklist RMBPC (*p* 0.02 within, *p* 0.006 between groups)	PEDro 5/11 RCT/PT, two-arm
+ Hars et al., 2014	Walking following piano, quick exercises/walking out of rhythmic patterns, *n* = 134 60 min, 1x/week, 25 weeks		HADS (*p* 0.924) non-significant decrease of depression	PEDro 10/11 RCT
# Im and Lee, 2014	Music therapy, art therapy, *n* = 34 60 min, 1x/week, 12 weeks	Autonomy and responsibility	GDS (*p* 0.000)	PEDro 10/11 RCT /PT, two-arm
# Jun et al., 2013	Music movement therapy, *n* = 40 60 min, 3x/week, 8 weeks	Integration function of physiological functioning	CES-D no sign. Change (*p* 0.280), but sign. Improvement mood POMS (*p* 0.040)	PEDro 11/11 PT, two-arm
# Kang et al., 2010	Warm-up hand exercises, MT art therapy, horticulture activity; MT: learn + listen songs, express emotion through motion and dance, later with instruments, n = 38 180 min, 6x/week, 3 weeks	Music free-flowing without direction to evoke past enjoyable memories	GDS (*p* 0.001)	PEDro 5/11 QE/PPT, non-equivalent control group
# Kim H. et al., 2016	Reminiscence, occupational, art, horticultural, music therapy: playing melodies/ accompaniment, *n* = 53 5x/week, 6 months	Encouragement to develop musical expression/imitate musical rhythms	GDS (*p* 0.09), not significant improvement	PEDro 10/11 RCT, compared pre-post trial
# Liu et al., 2014	Active and receptive (Chinese five-element music) music therapy, *n* = 50 60–120 min, 1x/week, 4 months	Integration of physiological functioning	HDRS post treatment (*p* < 0.05) and follow-up (*p* < 0.05)	PEDro 11/11 PT, two-arm
# Low et al., 2015	Training for home care providers, care workers, *n* = 189 champions 5 h, case manager 3 h care workers 4*2–3 h duration: 12 months		CSDD (*p* 0.0109) non- significant decrease of depression	PEDro 10/11 quasi-experimental design
* Magee et al., 2006	Singing (rituals welcome and goodbye, breathing exercise, vocal exercise, singing exercise, song singing), *n* = 1 60 min, one time	Wellbeing-processing, voice-training, songs	HADS, decreased depression, score changed from 9 to 3	COREQ29/32 CS, single
* Myskja and Nord, 2008	Music therapy (group, singing preferred songs, systematic pre-search of songs), *n* = 72 45 min, 2x/week, 11 weeks	Dementia symptoms, calming or activating, combined medical treatment, unclear	MADRS (*p* < 0.05)	PEDro 4/11 PS/PPT
# Onieva-Zafra et al., 2018	Music + reminiscence therapy together; musical experiences–listening/singing, *n* = 19 45 min, 2x/week, 8 weeks		GADI (*p* 0.01)	PEDro 5/11 PS/QE, nonrandomized
# Pongan et al., 2017	Choral singing, *n* = 59 12 weeks	Structure of singing, social context	GDS no sig. changes (p 0.68)	PEDro 10/11 RCT, multi-center
* Raglio et al., 2015	Active music therapy in one group, individual music listening in the other, *n* = 120–30 min, 2x/week, 10 weeks	MT followed the PWDs rhythm / music production to create nonverbal communication; built relationship by singing, using melodic + rhythmic instruments (impro), facilitated expression / modulation of PWD's emotion, promoted affect attuned moments	Non-significant decrease to SC (all with significant CSDD decrease)	PEDro 10/11 RCT, multi-center
* Ray and Mittelman, 2017.	Music and movement, singing, tonal activities, *n* = 132 15–60 min, 3x/week, 2 weeks	Work on arising themes	CSDD (*p* 0.001)	PEDro 5/11 QS
+ Reychler et al., 2015	Listening to ambient music, 120 beats/min, *n* = 41 75 min, 3x/week, min. 4 weeks		The Borg Scale of Perceived Exertion, no difference (*p* 0.02)	PEDro 10/11 RCT, crossover
+ Sánchez et al., 2016	Individualized music sessions, according to musical preferences, *n* = 22 30 min, 2x/week, 16 weeks		CSDD (*p* = 0.006)	PEDro 10/11 RCT
# Särkämö et al., 2016	Singing or music listening by coached caregiver, *n* = 84 90 min, 1x/week, 10 weeks		CBS: decrease of depression more in mild dementia by music listening and singing, depend on groups *p* 0.001–0.79	PEDro 10/11 RCT
# Thomas et al., 2017	Music and Memory-program: play lists tailored to personal history and preferences, *n* = 196 180 days, daily		PHQ-9 (*p* 0.53), no reduced depression	PEDro 5/11 PPT, controlled trial
+ Travers and Bartlett, 2011	radio program “Silver Memories” 1920–1950, *n* = 113 60 min, daily, 3 months		GDS (*p* 0.003)	PEDro 4/11 MM, scales and interview
+Verrusio et al., 2014	Music listening (Jazz, Classical, Modern = 3 sub-groups)) + physical exercise training, *n* = 24 60 min, 2x/week, 24 weeks		GDS (*p* 0.01)	PEDro 10/11 PS/RCT
# Wang et al., 2017	Kagayashiki music care: activities designed within rehabilitation + music, musical activities and/or physical activities are carried explicitly, *n* = 149 30 min, 2x/week, 24 weeks	One's perceived self-efficacy can result increasing self-confidence + success in executing a given task	CSDD no significant decrease (*p* 0.190)	PEDro 6/11 QE/LS
* Werner et al., 2017	Group singing, receptive music therapy, instrumental improvisation, dance/movement, *n* = 117 40 min, 2x/week, 10 weeks	Validation, expression of feelings, and memories, shared experiences	MADRS (*p* 0.000) Cohen's d = 0.49	PEDro 9/11 PT
# Yap et al., 2017	Percussion instruments, free play, *n* = 31 60 min, 1x/week, 10 weeks		GSD (*p* 0.496) non-significant	PEDro 10/11 PS, randomized cross-over pilot study

***Key:** Two-arm: an intervention involving only standard care and a music intervention, without a third condition comparator*.

*BDI, Beck Depression Inventory; CBS, Cornell-Brown Scale for Quality of Life; CES-D, The Center for Epidemiologic Studies Depression Scale; COD, crossover design; COMP, A composite depression scale constructed on the HAM-D (double weighted), BDI, and HADS-Dz-scores; CSDD, Cornell Scale for Depression in Dementia; GADI, Goldberg Anxiety and Depression Inventory; GDS, Geriatric Depression Scale; HADS, Hospital Anxiety and Depression Scale; HADS-D, Hospital Anxiety and Depression Scale- Depression sub-scale; HDRS, Hamilton Depression Rating Scale; HAM-D, Hamilton Rating Scale for Depression; MADRS, Montgomery-Asberg Depression Rating Scale; PHQ-9, Patient Health Questionnaire; POMS, Profile of Mood States; PWDs, Persons with dementia*.

**Intervention coding:** * intervention delivered by trained or certified music therapist; # intervention delivered by other professional; and + unclear.

***Design codes:** CS, case study; CDA, collaborative discourse analysis; LA, longitudinal study; OS, observational study; PE, pre-experimental; PPGD, prospective, parallel-group design; PPT, pre and post-test; PS, pilot study; PT, pragmatic trial; QE, quasi experimental; QS, qualitative study; RCT, randomized control trial; RCS, retrospective cohort study; SSR, single subject research*.

Sample sizes ranged between 1 and 12.576, with a mean sample of 379. Only six of the 41 studies included follow ups, undertaken after 3 or 6 months, all amongst most recent studies (Coulton et al., 2015; Kim H. et al., 2016; Sánchez et al., 2016; Särkämö et al., 2016; Pongan et al., 2017; Ray and Mittelman, 2017).

The quantitative studies scored between 4 and 11 on PEDro, and the one qualitative study met 29 of 32 elements in COREQ. Twelve interventions were led by a music therapist, 23 led by another professional; and six by a leader of unknown training. Twenty-six studies had significant findings: nine of these were led by a music therapist, twelve by another professional, and a further five had a leader of unknown training. Fifteen of the 26 music studies with significant findings were assessed as having high quality findings (8–11/11 on PEDro). The other six studies were assessed as being of low quality, with missing elements including blinding, similarity at baseline, concealed allocation, and clear randomization.

### Interventions

Programs offered were sessions of between 15 and 360 min, with 30–60 min being most common; held between once weekly and daily, most often weekly or twice per week; for periods from one day to 24 months, most often 10–12 weeks.

Very different methods and intervention types were included in treatment. Most frequently reported interventions in the 26 studies with successful outcomes were receptive music therapy and music listening (17). In nine studies, mixed intervention types (between two and five types) were included in treatment. Instrumental play and improvisation were also utilized in nine studies. In eight studies, individualized individual preferred music or reminiscence were also included with music listening or singing. In six studies, the intervention type was singing, and music movement was included in six studies as well. Activities were selected due to theoretical models or findings of therapeutic processes or mechanisms of change described in the following paragraphs.

### Therapeutic Processes

Therapeutic processes described in the music studies were closely linked to mechanisms of change. These processes focussed on physical activation, processing of emotion and social relationships. Physical activation was seen to be activated through rhythmic patterns (including march rhythms in 4/4 time), physical reaction to emotions and improvement of movement. Processing of emotions supported changes from feelings of anger and fear to increased positive emotions and emotional responses including stimulation of happy memories, subjects' interests, preferred autobiographical music, and musical interests, and enjoyment through activation of the limbic and paralimbic systems. Processes relating to social relationships are described as interaction through playing of instruments, promotion of empathic relationships, increased communication, and reduced social isolation.

### Proposed Mechanisms of Change

The studies reviewed suggest that shifts in depressive symptoms resulting from engagement in music and music therapy are a result of mechanisms of change across several domains:

- Physical: neurophysiological and neurochemical effects, such as endorphin release, stimulation of cognitive functioning i.e., reminiscence and activation of amygdala, hippocampus, and nucleus accumbens;
- Cultural (creative/aesthetic): processing of music stimuli in rhythm and pitch, musical experience including movement, physiological functioning, and imitating of musical rhythm;
- Intrapersonal: improvement of well-being, activation of remaining capabilities, self-efficacy, validation, increase of autonomy, and self-confidence developed through experiences of success;
- Social: improvement of interaction skills and relational abilities, to trigger interpersonal relationship, to stimulate social participation.

## Discussion

This review reveals significant differences between the creative arts modalities with respect to research quantity, type and quality. The number of studies about music interventions (*n* = 41) was significantly more than other modalities, i.e., art (*n* = 17), dance (*n* = 13), and drama (*n* = 4). Types of research were clearly distinct between modalities, with 40 of the 41 music studies, and all 13 dance studies being quantitative research, while art studies included predominantly quantitative, but also qualitative and mixed-methods studies, and the small number of drama studies involved qualitative, mixed-methods, and quantitative approaches. This indicates a need for more research in the creative arts therapy modalities that are as yet under-represented in the literature. While 13 dance studies were included in this review, none of those interventions was actually led by a dance movement therapist, so the need for more research in dance movement and drama therapy is particularly evident.

Quality issues differed between the modalities. Art therapy studies were found to be of medium quality with the main issues in quantitative studies being small sample size, a general lack of generalizability and a lack of rigorous efforts to ensure validity in the findings. Issues in qualitative studies in art therapy also relate to a lack of rigor to ensure creditable data analysis and inadequate reporting in data collection. This differs somewhat to the dance movement studies, which were largely RCTs and scored across the range from low to high on the PEDro scale roughly evenly. Quality issues for this modality relate to the lack of actual DMT interventions. Dance was the only modality for which this was an issue. Drama studies scored middle to high, with quality relatively high for quantitative studies (8 and 9/11 on PEDro), but lower for qualitative studies (19 and 21/32 on COREQ). Music studies scored high more consistently. Very few of the studies included follow up: art (0); dance 4/13; drama (0); and music 6/41.

The sections to follow discusses some of the most salient findings about each modality.

### Art

The choice of art media that is culturally relevant to the participant was repeatedly found to be a key factor of engagement. The fact that art interventions examined were at least 12 weeks long indicates that longer-term approaches might be most effective with this population. In consideration of the many mental and physical challenges faced by older adults, longer-term approaches might be more congruent with relationship building and sustaining the outcomes of art therapy. The effectiveness and impact of short-term programs remains to be studied. Future studies might also examine how personalized use of traditional art media might contribute to client outcomes. The quality of most art intervention studies was scored as medium to low (based on PEDRO and COREQ scores) with few studies effectively randomizing participants and/ or providing adequate details in the methodology.

### Dance

The dance studies primarily focussed on the learning of dance steps and sequences, and repetition of these across sessions. Dance movement therapy, in contrast, often does not include the learning of structured steps, but rather prioritizes more improvised and expressive dance experiences. However, the effectiveness of learning of steps for amelioration of depression and its symptoms evidenced in these studies may point to it being a worthwhile consideration for DM therapists, especially for this older adult age group. One issue arising in the dance studies was the perception by participants and possibly program hosts that dance interventions are more suitable for women. As the rate of depression is the same for men and women, and its functional impacts are greater for men, this indicates a challenge, with an effective modality not potentially being considered by 50% of the people impacted by depression.

The quality of dance studies was varied, with ratings evenly distributed from the lowest to highest PEDro scores. Lower scoring studies were often missing elements of blinding of subjects, therapists, and assessors, and concealment of allocation.

### Drama

While few in number, the three studies involving drama therapists indicated that positive outcomes could be achieved in programs of 12 weeks in length. The findings of these studies were consistent with recent literature in drama therapy emphasizing the benefits derived from opportunities for playful interaction and the externalization of significant experiences through drama; these processes were found to reinforce internal resources and contribute to a sense of generativity (see Jennings, 2018).

### Music

Twenty-six of the 41 music studies demonstrated significant findings in treatment of depression and depressive symptoms. The most effective interventions were provided by trained music therapists, with nine of the twelve studies involving these professionals having significant findings, whereas only twelve of the 23 studies involving other professionals had significant findings. Thus, interventions led by music therapists appeared more suitable than those led by other professionals for treatment for depression and for depressive symptoms of older adults. Effective interventions were diverse and included receptive music therapy and music listening, mixed intervention types, instrumental play and improvisation, and individualized, individual preferred music or reminiscence in music listening or singing.

### Methodological Issues

We began this review with the intention of exploring the effects of creative arts interventions on older adults experiencing depression, as well as relationships documented between intervention activities, therapeutic processes, and mechanisms seen to lead to outcomes. However, this second task proved not to be straightforward, with studies often not providing adequate (or any) discussion about processes or mechanisms that were expected or elicited through interventions. In articles that did discuss processes or mechanisms, claims were largely not substantiated with data. Other studies appeared to have been predicated on theories of change about outcomes expected from activities and associated therapeutic processes but did not explicate these. This was particularly evident in the dance studies, where there was much focus on physical movement, without specific articulation of the well-evidenced relationship between physical exercise and reduced depression or depressive symptoms.

This finding gives rise to a consideration that we did not assess one important quality point, that of the quality of interventions. Given that the quality of an intervention is likely to significantly impact findings, this would seem an appropriate and relevant process. While we scored the methodological quality of studies using COREQ and PEDro tools, this process did not offer any insight about how well-considered interventions appeared, how well substantiated they were from theory or evidence, or whether justification for activities or processes to be employed was adequate. Thus, an additional quality process we recommend would be for the assessment of the quality of choices made and processes employed. The use of manualised interventions or descriptions of clear intervention protocols may contribute to better practice in this respect.

Another methodological challenge was the lack of discussion in the studies examined of the relationship between expected outcomes and specific symptoms of depression as identified in DSM-V or ICD manuals. We had initially tried to align outcomes of studies with these formally identified symptoms, but few studies specifically mentioned addressing these. We recommend that future studies pay specific attention to identified symptoms of depression.

One risk of bias in this study is the fact that all authors are creative arts therapists. We attempted to minimize bias caused by this factor by ensuring that at least two authors were involved in extracting data from each study and that they double checked each other's work. Other strategies we employed for reducing bias were the inclusion of all studies, those with significant and non-significant findings, and adherence to strict inclusion criteria.

An additional limitation for research that was considering creative arts broadly was that our study did not include studies on writing, or the broader categories of expressive and creative arts that were not modality specific. Future research may be best to include these topics.

### Recommendations for Further Research

Our first recommendation for future research is for studies that meet quality standards for both quantitative or qualitative approaches, given that so many of the studies we examined did not. Our findings indicated a significant need for well designed, detailed studies of the impact of all creative arts therapies in the targeted treatment of depression in older adults.

We recommend that future studies include more specific focus on *how* interventions work, as well as *if* they work, given the under-development of theories of change about how creative arts interventions are seen to be effective in addressing depression in studies reviewed, and the lack of clear explication and testing of processes and mechanisms considered to contribute to therapeutic outcomes. In addition, the differences between, and sequencing of, individual and group interventions remain understudied.

Creative arts therapies are increasingly being offered as part of a range of complementary therapies in integrative care settings. However, no studies examined their cost effectiveness, either comparing creative arts modalities with each other, or between CA modalities and other therapeutic approaches. This prompts a recommendation for future inquiry, which would increase understanding of how creative arts interventions, including CA therapies, might be utilized as psychosocial prescriptions to increase effectiveness and reduce costs of healthcare of older adults. Increased collaboration between creative art therapy researchers could also be useful for improving research outcomes. Future studies could also examine how new technology like virtual reality and telehealth might contribute to the potential of creative arts interventions for the health of older adults.

### Implications for Practice

Given our findings that interventions led by certified creative arts therapists resulted in more significant or positive outcomes than interventions led by other professionals, we recommend that interventions for depression with older adults be provided by certified creative arts therapists. Because relatively few interventions included developed theories of change about interventions chosen, processes implemented and the expected relationship with these and changes in depressive symptoms, we consider that clinical practice may be similarly improved with better articulation of all of these considerations in the planning and delivery of interventions for depression.

## Conclusion

This review examines evidence for the effects of creative art interventions on depression and depressive symptoms of older adults. The majority of 51 of the 75 studies examined demonstrated either significant quantitative or positive qualitative findings (12/17 of art, 10/13 of dance, 4/4 of drama and 26/41 of music, and music therapy). The quality assessment of these studies differed between disciplines, with medium quality in art studies, the full range from low to high in dance studies, middle to high in in drama therapy, and high in the majority of studies with significant findings in music. Certified art therapists were involved in the majority of studies with significant findings: in art, 8/12; in drama, 3/4; and in music, 9/12 studies involving music therapists and 12/23 studies involving other professionals. No studies involving DMT fitted this criterion.

Mechanisms of change gleaned from the studies include physical (improvements in balance, muscle strength; neurochemical effects, such as endorphin release), intra-personal (positive views of self; strengthened agency and mastery; communication and processing of emotions; coping strategies), cultural (creative expression, aesthetic pleasure), cognitive (stimulation of memory), and social (increased social skills and connection) elements that were all considered to be causal in reduced depression and symptoms. Recommendations for future research includes stronger focus on trialing of processes and mechanisms, considerations of the value of short vs. longer term therapy, and cost-effectiveness of creative arts therapy modalities in comparison with each other as well as with other type of therapeutic treatment.

## Author Contributions

FB, GK, NS, TW, and KD conceptualized the project and designed the study. KD co-ordinated the project, and led writing of introduction, method and discussion sections and integrative analysis. KC-H, ED, JE, ME, KM, and OS undertook the data gathering, first stage of analysis and data entry for sections on art therapy, dramatherapy, dance movement therapy, and music therapy respectively. GK, KM, NS, ED, KD, FB and TW undertook the second phase of analysis and write up of results for sections on art therapy, drama therapy, dance movement therapy and music therapy respectively. All authors contributed to manuscript revision and read and approved the submitted version. Authors are listed alphabetically except first three authors Dunphy, Baker, and Dumaresq.

### Conflict of Interest Statement

The authors declare that the research was conducted in the absence of any commercial or financial relationships that could be construed as a potential conflict of interest.

## Abbreviations

AT: art therapy
DMT: dance movement therapy
DT: drama therapy
MT: music therapy
LLD: late life depression
MDD: major depressive disorder.
